# Homer2 and Homer3 Act as Novel Biomarkers in Diagnosis of hepatitis B virus-induced Hepatocellular Carcinoma

**DOI:** 10.7150/jca.52118

**Published:** 2021-04-19

**Authors:** Ping Luo, Chunzi Liang, Wei Jing, Man Zhu, Hu Zhou, Hongyan Chai, Paul F. Worley, Jiancheng Tu

**Affiliations:** 1 Department of Hematology, Zhongnan Hospital of Wuhan University, Wuhan, China.; 2 Department of Clinical Laboratory, Key Laboratory of Laboratory Medicine of Henan, the First Affiliated Hospital of Zhengzhou University, Zhengzhou 450000, China.; 3 Department of Medical Laboratory, The Central Hospital of Wuhan, Tongji Medical College, Huazhong University of Science and Technology, Wuhan, Hubei 430014, P.R. China.; 4 Department of Transfusion, Tongji Hospital, Tongji Medical College, Huazhong University of Science and Technology.; 5 Department of Neuroscience, School of Medicine, The Johns Hopkins University, Baltimore, MD 20205, USA.; 6 Department & Program of Clinical Laboratory Medicine, Center for Gene Diagnosis, Zhongnan Hospital of Wuhan University, Wuhan 430071, China.

**Keywords:** Homer2, Homer3, HCC, Tumorigenesis, Diagnosis.

## Abstract

**Background:** Hepatocellular carcinoma (HCC) is one of the most common causes of cancer‐related mortality worldwide. Early detection of HCC can significantly improve patients' outcomes. An increasing number of studies have validated that Homer is dysregulated in cancers and may serve as diagnostic markers. In the present study, we investigated the expression profile and diagnostic significance of Homer2 and Homer3 in hepatitis B virus-induced HCC (HBV-HCC).

**Methods:** Quantitative real-time PCR (QRT-PCR), western blot analysis and immunohistochemistry analysis.

**Results:** Homer2 and Homer3 were downregulated in HCC. The expression of Homer2 was associated with tumor differentiation grade (*P*= 0.012) and total protein (TP) level (*P*= 0.032). Homer3 was related to tumor size (*P*= 0.010), tumor nodes (*P*= 0.026) and γ-glutamyl transferase (GGT) level (*P*= 0.001). The receiver operating characteristic curve analyses indicated that the combination of Homer2, Homer3 and AFP possessed a high accuracy (AUC=0.900) to diagnose HCC cases from healthy controls.

**Conclusion**: Our data indicated that Homer2 and Homer3 were downregulated in HCC and might be potential diagnostic marker for HCC.

## Introduction

The mortality of Liver cancer ranks second among human cancers worldwide 1, 2. It is reported that hepatocellular carcinoma (HCC) occupy approximately 80% of liver cancers 3. In 2018, about 840,000 HCC patients were newly diagnosed and 782,451 deaths were reported. Furthermore, it is estimated that by 2030, the new cases of HCC may reach to 1 million 4. Among many reported risk factors of HCC, cirrhosis caused by chronic hepatitis B or C virus infections is the major one 5-7. Data has shown that the 5-year-survival rate of HCC patients remained less than 12% due to the delayed diagnosis 8. Though early diagnosis, together with interventional therapy and/or surgical methods can significantly improve the outcome of HCC, unfortunately the majority of HCC patients are diagnosed at an advanced stage 3. Currently, serum alpha-fetoprotein (AFP) levels combined with computed tomography (CT) and magnetic resonance imaging (MRI) are the most common non-invasive diagnostic methods, but all of them have sensitivity problems 9. Hence, new non-invasive biomarkers with high sensitivity and specificity for the early diagnosis of HCC, especially among high-risk people such as patients with the chronic liver disease would be highly beneficial.

Homer protein families in mammals are mainly made up of three members, Homer1, Homer2 and Homer3, each of them has several isoforms due to alternative splicing 10, 11. It is reported that all Homer family members have a common EVH1 domain at the N-terminus 12. In 2008, Derek and his colleagues found that Homer3 was over expressed in Acute Myelocytic Leukemia (AML) samples with favorable cytogenetics 13. In 2016, Shen et al. revealed that Homer3 were overexpressed in esophageal squamous cell carcinoma, and significantly linked with pN and pStage 14. In 2017, Sun et al. 15 revealed that the expression of Homer2 was increased in rectal carcinoma (RC) via co-expression network analysis. They also found that Homer2 expression was correlated with higher pathological stage and shorter overall survival of RC patients. Recently, another study concluded that low-grade endometrioid endometrial adenocarcinoma patients with high expression of Homer2 protein had a better outcome 16. Thus, we hypothesize that Homer plays an important role in the process of tumors. Our previous research has revealed that Homer1, downregulated both in the HCC cell line & tissues, can be used as a diagnostic maker for HBV-HCC 17. Toward this direction, we assumed an association between Homer2, Homer3 and HCC. Huang et al. 18 found that Homer2 and Homer3 can negatively regulate the activation of T cell through competing with calcineurin and by binding of nuclear factor of activated T cells (NFAT). T cell associated immune response were related to the survival of HCC 19, which further supported our hypothesis.

Here, we aim to evaluate the expression level of Homer2 and Homer3 & its correlation with clinicopathological characteristics of patients with HBV-HCC, and then to explore the value of novel biomarker of Homer2 and Homer3 in the diagnosis of HBV-HCC.

## Materials and Methods

### Sample collection

A total of 77 HCC (71 men and 6 women, mean age 58±10) tissues and paired adjacent non-tumor tissues were collected from HCC patients who underwent surgical resections from October 2014 and October 2019 at Zhongnan Hospital of Wuhan University. At the same time, 183 patients' whole blood samples from the same hospital during 2016 were collected. Patients were classified into three group: 72 patients with HCC (59 males and 13 females, mean age 57±13), 52 patients with hepatitis B (37 males and 15 females, mean age 52±14), and 59 the cirrhosis (49 males and 10 females, mean age 56±12), Meantime, 109 healthy blood samples (80 males and 29 females, mean age 54±11) were collected from the Physical Examination Center. All of the patients were selected based on medical or pathology reports. The inclusion criteria were as follows: HBsAg positive and without any other liver diseases, such as alcoholic, autoimmune, hepatitis C and metabolic liver diseases; newly diagnostic. Exclusion criteria were as follows: patients with history of chemotherapy or radiotherapy; patients with no comprehensive clinical data. The species were stored at -80˚C until RNA extraction.

### RNA extraction and cDNA synthesis

Total RNA was extracted using Trizol reagent (Invitrogen, Carlsbad, CA, USA) as described by the manufacturer. After extraction, RNA concentration was measured using Nanodrop 2000 spectrophotometer (Thermo Scientific Inc., Waltham, MA, USA). The reverse transcription (RT) reaction of 1μg RNA was performed with random primers in a final volume of 20 μL using PrimeScriptTM RT reagent Kit with gDNA Eraser (Takara, Japan). Reverse transcription conditions were as follows: 42°C for 2 min, and then 37°C for 15 min, 85°C for 5s, followed by storage at 4°C. All cDNA samples were stored at -80°C before real-time PCR analysis.

### Real-Time Polymerase Chain Reaction

Real-Time PCR was performed on Bio-Rad CFX96 (Bio-Rad Laboratories, Inc., Hercules, CA, USA) using SYBR-Green I Premix Ex Taq following manufacturer's instructions. The reaction was carried out in a volume of 20μl containing 1.5μL cDNA, 10μL SYBR Green mix, 1.6μL gene-specific forward and reverse primers (10μM), and 6.9μL nuclease-free water. Glyceraldehyde-3-phosphate dehydrogenase (GAPDH) was selected as housekeeping gene for normalization. The primers for the reaction were as follows: Homer2 sense: 5'-TCACCGTTTCCTACTTCTATG-3' and antisense: 5'-CCTGCGTCTTGTCTT-TGG-3'; Homer3 sense: 5'-CGCACTCACTGTCTCCTATT-3' and antisense: 5'-GGAACTTCTCGG-CAAACT-3'; GAPDH sense: 5′-AGAAGGCTGGGGCTCATTTG-3′ and antisense: 5′-GCAGGAGGCATTGCTGATGAT-3′. The amplifications started at 95°C for 5 min followed by 40 cycles, each consisting of denaturing for 30 s at 95°C, annealing for 30 s at 62°C, and elongation for 30 s at 72°C. All reactions were amplified in duplicate with no-template controls included. Amplification specificity for each gene was confirmed by a single distinct melting curve. PCR products were separated using 2.0% agarose gel electrophoresis to confirm the presence of a single band at the expected amplified size.

### Cells and cell culture

The normal human hepatocyte cell line L-02 were bought from the Procell Inc. (Wuhan, China). HCCLM-9 were maintained at our laboratory. We purchased human HCC cell lines HepG2 from the China Center for Type Culture Collection (CCTCC, Wuhan, China). All the three Cells were cultured in RPMI 1640 medium (Gibco, USA) containing 10% fetal bovine serum (FBS, Gibco, USA), 50 U/ml penicillin and 50 U/ml streptomycin.

### Western blot analysis

The stable cells were lysed using RIPA lysis buffer in the presence of PMSF proteinase inhibitor (Beyotime, China). Approximately a total of 40 ug protein was electrophoresed on 12% sodium dodecyl sulphate polyacrylamide gel and transferred onto polyvinylidene difluoride (PVDF) membrane. The membrane was blocked with 5% non-fat milk in PBST before incubation with the primary antibody. The primary antibodies used for Homer2 and Homer3 were mouse polyclonal (Abcam, USA). GAPDH was used as a loading control. After incubated with secondary antibody for 1 h at room temperature, the signals were visualized with enhanced chemiluminescence (ECL) (Beyotime, China).

### Immunohistochemistry analysis

Cells were seeded in 6-well plates, fixed in methanol for 15 min, and then permeabilized with 0.2% Triton X-100 for 15 min. Endogenous peroxidase activity was blocked by incubating in 3% H_2_O_2_ solution. Next, the cells were blocked with goat serum for 30min and incubated with Homer2 and Homer3 antibodies (1:100, Abcam, USA) overnight at 4 °C. Subsequently, samples were incubated with the corresponding HRP-conjugated secondary antibody solution for 45min. And then DAB (DAB-0031; Fuzhou, China) substrate solution was applied for antibody staining for 10min. Nuclei were stained with hematoxylin.

### Statistical Analysis

We presented normally distributed data as mean ± standard deviation (SD) and skewed data as median and inter-quartile range. The Shapiro-Wilk test was carried out to check the normality of the distribution. For normally distributed data, student's t test was used to compare 2 groups of continuous variables and one-way ANOVA was used for the comparison among multiple groups, while non-normally distributed data was analyzed by Kruskal-Wallis variance analysis. Qualitative data was tested using Chi-square test. To evaluate the diagnostic value of Homer2 and Homer3, receiver operating characteristic (ROC) curves were made, and the area under the ROC curves was determined. The statistical analyses were performed with the SPSS 17.0 software (SPSS, Chicago, IL, USA) and GraphPad Prism 5 software (GraphPad software, La Jolla, CA, USA). All statistical tests were two-sided and *P*<0.05 was considered statistically significant.

## Results

### Homer2 and Homer3 expression in HCC cell lines

**Figure 1:**
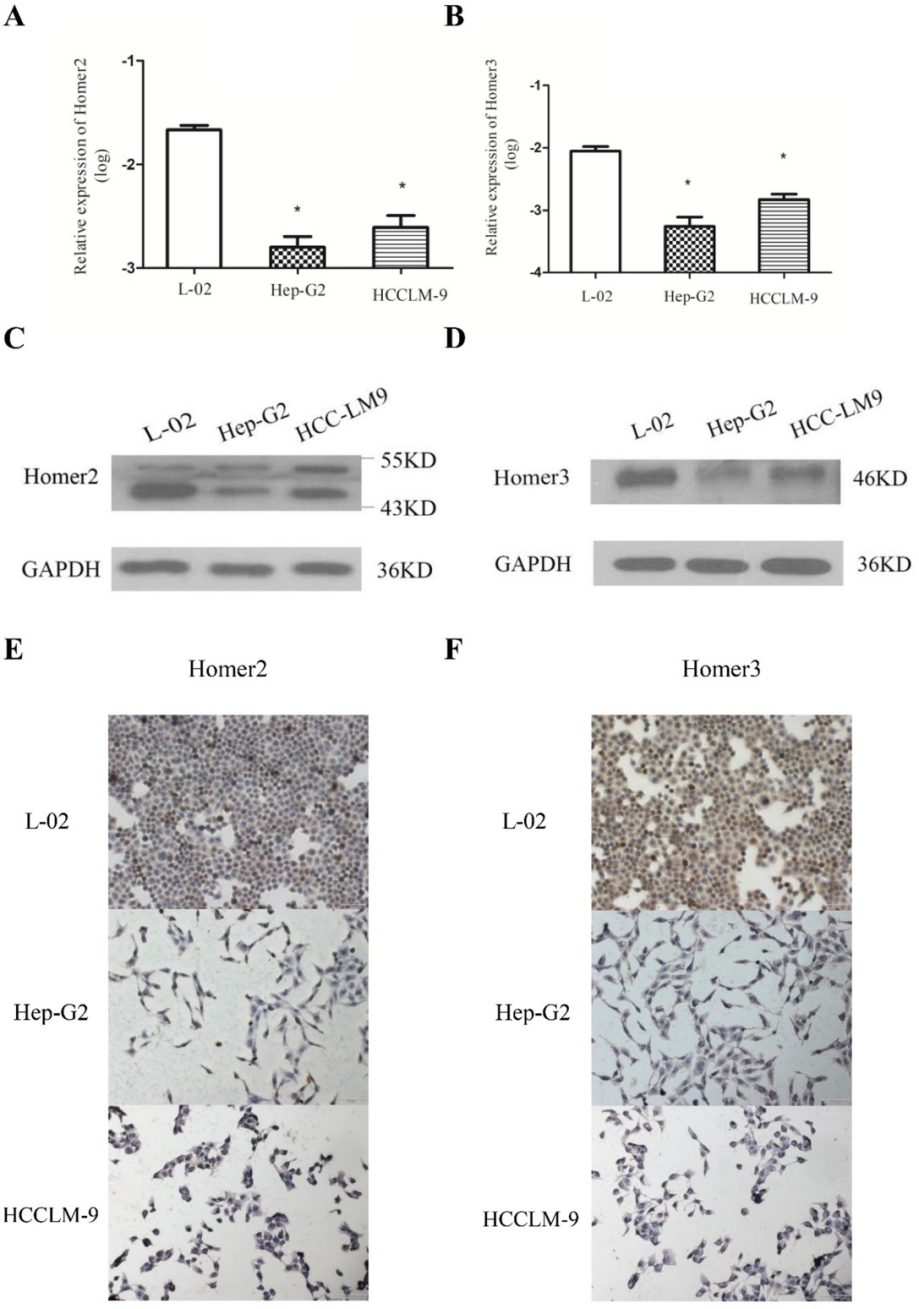
** Evaluation of Homer2 and Homer3 expression in HCC cell lines**. Expression of Homer2 in HCC cell lines were lower than that in L-02 cell **(A, C, E).** Expression of Homer3 in HCC cell lines were lower than that in L-02 cells **(B, D, F).** * *P*< 0.05.

QRT-PCR, Western blot analysis and immunohistochemical method were used to detect the expression of Homer2 and Homer3 in several cell lines (L-02, Hep-G2, and HCCLM-9). We found that both Homer2 and Homer3 expression were significantly higher in L-02 cell line than in other cell lines (Hep-G2 and HCCLM-9) in RNA and protein level (Figure 1).

### Homer2 and Homer3 were down-regulated in HCC tissues

**Figure 2:**
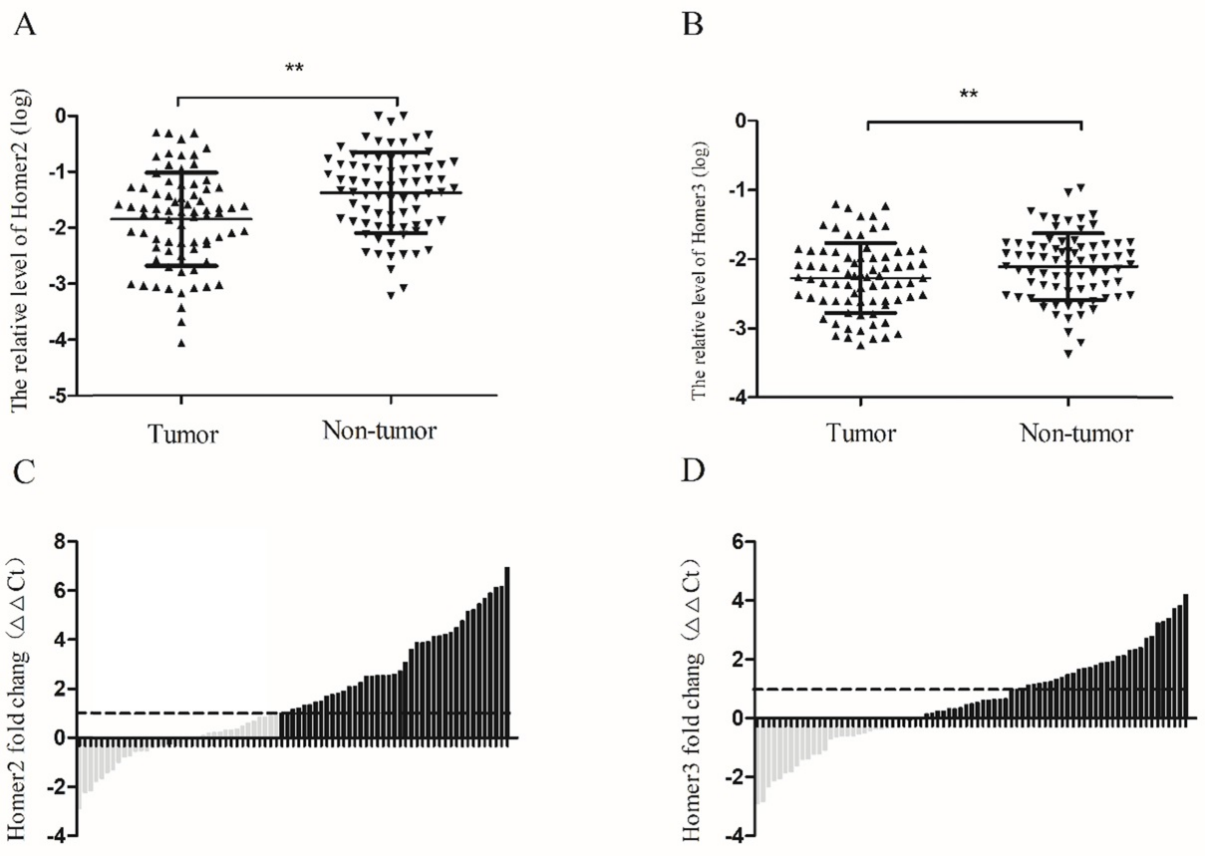
Homer2 and Homer3 expression in HCC tissues. The relative Homer2 and Homer3 expression were determined by RT-qPCR. Δ Ct values were calculated by subtracting the GAPDH Ct value from the Homer2 or Homer3 Ct value. Smaller ΔΔ Ct value indicates higher expression. ΔΔ Ct = (Ct _Homer2/Homer3_-Ct_ GAPDH_) of HCC - (Ct_ Homer2/Homer3_- Ct_ GAPDH_) of NT]. (NT: paired noncancerous tissues of HCC). (**A**) Levels of Homer2 in tumor tissues are significantly lower than that in non-tumor tissues. (**B**) Levels of Homer3 in tumor tissues are significantly lower than that in non-tumor tissues. (**C**) Homer2 was reduced by at least twofold in 53.2% (41/77) of the HCC tissues. (**D**) Homer3 was downregulated in 62.3% (48/77) of HCC tissues. All data were analyzed using Student's t test. Data were presented as mean ± SD, * *P* < 0.05, ** P< 0.01.

RT-qPCR was used to detect the expression levels of Homer2 and Homer3 in HCC tissues and paired normal liver tissues derived from 77 HCC patients. Our results revealed that the relative expression levels of Homer2 and Homer3 were down-regulated in HCC tissues compared with matched adjacent tissues. (Homer2: *P*<0.01,** Figure 2A**; Homer3: *P*<0.01, **Figure 2B**). Then, waterfall plot demonstrated that Homer2 was reduced by at least twofold in 53.2% (41/77) of the HCC tissues (**Figure 2C**). We also found that Homer3 was down-regulatedin 62.3% (48/77) of HCC tissues (**Figure 2D**).

### Clinicopathological data analysis of HCC patients

The detailed clinic parameters of enrolled 77 HCC tissue patients and clinicopathological relevance analysis were summarized in **Table 1**. Of all the 77 patients, 71 are male and 38 patients are over the age of 55. As to the severity of the tumor, 9 patients are low differentiation, 47 patients with tumor size bigger than 5cm, 29 patients belongs to TNM Ⅲ~Ⅳ and 8 patients had multi tumor nodes. The number of patients with AFP, GGT higher than normal value is 32, 42 and the numer of patients with TP and Alb lower than normal value are 22, 12. Correlation analysis results showed that Homer2 expression was significantly associated with tumor differentiation grade (*P* = 0.012) and TP (*P* = 0.032), while the expression of Homer3 was significantly related to tumor size (*P* = 0.010), tumor nodes (*P* = 0.026) and GGT level (*P* = 0.001). Thus, we hypothesized that down-regulation of Homer2 and Homer3 might play an important role in the development of HCC.

### Homer2 and Homer3 level in peripheral blood among subgroups

**Figure 3:**
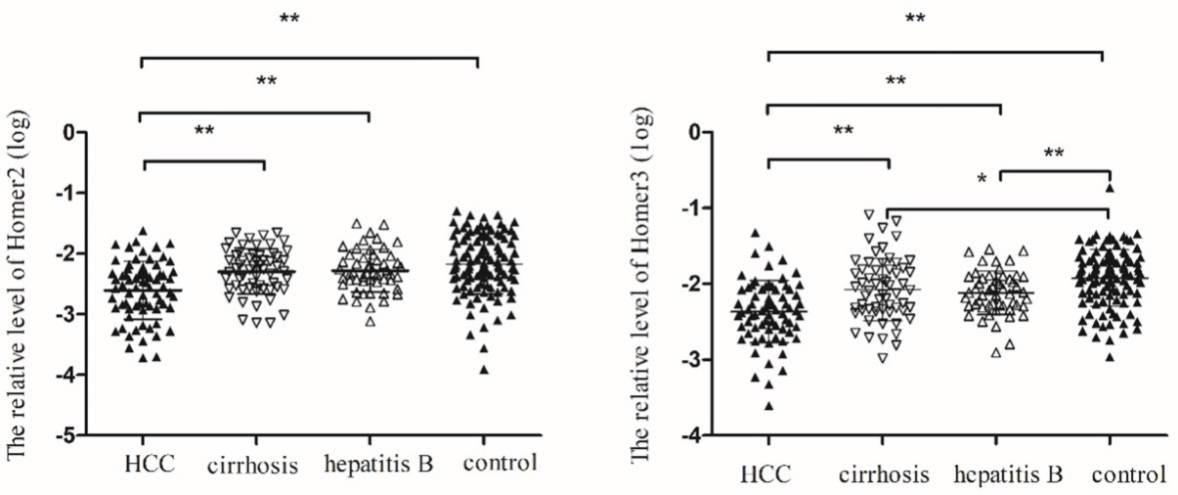
Homer2 and Homer3 expression in peripheral blood among subgroups. (**A**) Homer2 expression in HCC was lower than that in cirrhosis, hepatitis B and the controls. No differences were observed among other subgroups. (**B**) Homer3 expression in HCC was lower than that in cirrhosis, hepatitis B and the controls and Homer3 expression in cirrhosis and hepatitis B were lower than that in controls. No differences were observed between hepatitis B and cirrhosis. Data were analyzed using oneway ANOVA. * *P* < 0.05, ** *P* < 0.01.

We tested the expression of Homer2 and Homer3 in peripheral blood in 72 HCC patients, 59 cirrhosis patients, 52 hepatitis B, and 109 control cases. **Table 2** showed the main demographic and clinical characteristics of studied subjects**.** No difference was observed in risk factors including gender, age, smoking, alcoholism, whereas we found a significant difference in ALT, AST, TBIL, GGT and GLU among the groups. As illustrated in **Figure 3**, we found that Homer2 expression differed significantly between HCC and cirrhosis (*P*< 0.01), hepatitis B (*P* < 0.01), and the controls ((*P*< 0.01). For Homer3, its expression in HCC was lower than that in cirrhosis (*P* < 0.01), hepatitis B (*P* < 0.01) and the controls (*P* < 0.01) and Homer3 expression in cirrhosis (*P* < 0.05) and hepatitis B (*P* < 0.01) were lower than that in controls.

### Homer2 and Homer3 increased after operation in HCC

**Figure 4:**
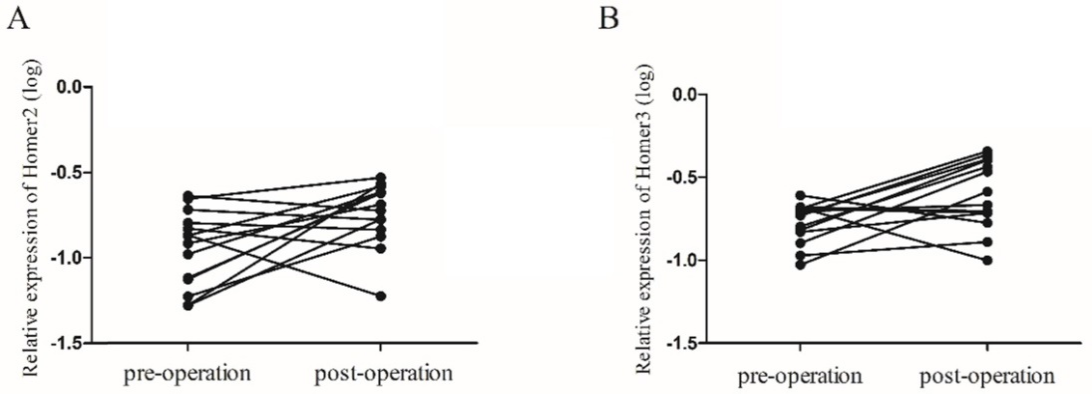
The expression levels of Homer2 and Homer3 in 14 paired preoperative and postoperative peripheral blood samples. (**A**) Levels of Homer2 was increased 2 weeks after surgical (*P*=0.024); (**B**) Levels of Homer3 was increased 2 weeks after surgical (*P*=0.022).

We next explored whether peripheral blood expression level of Homer2 and Homer3 could be used to monitor tumor dynamics. 14 paired cases of HCC patients were enrolled to compare the Homer2 and Homer3 expression levels between preoperative and postoperative samples. We found that the Homer2 level increased in 9 of 14 HCC patients (64.3%), and the Homer3 levels rose in 10 of 14 HCC patients (71.4%), approximately 2 weeks after surgery (**Figure 4**).

### Diagnostic value of Homer2 and Homer3

**Figure 5:**
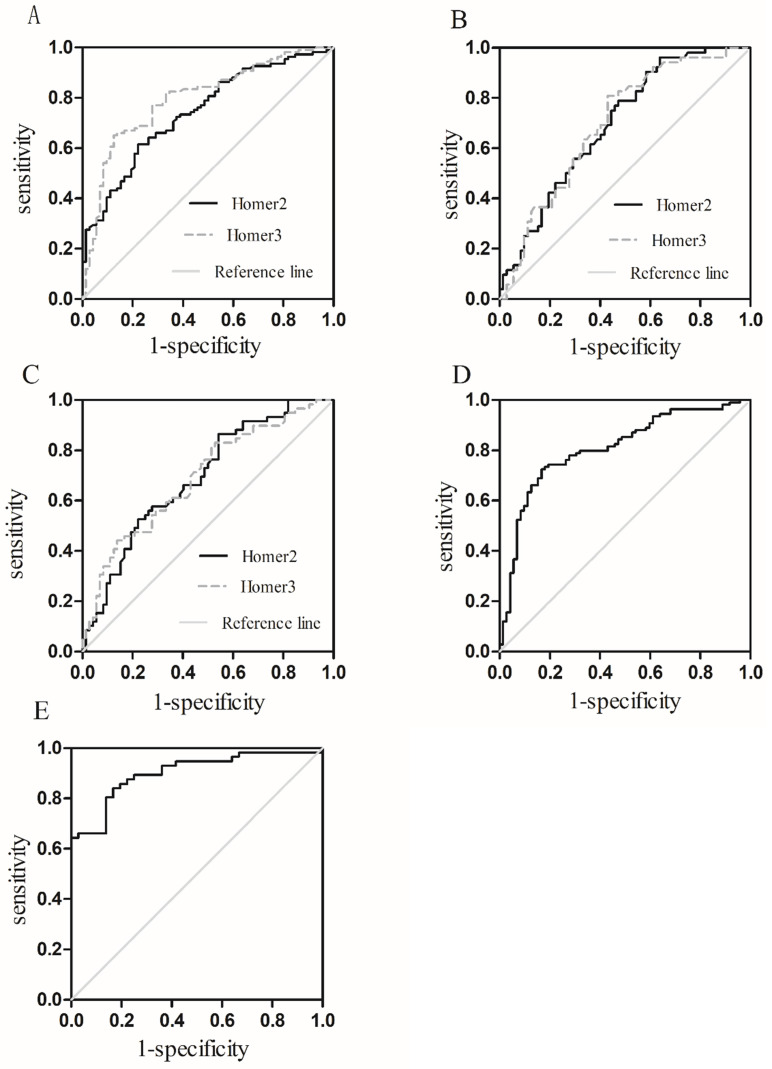
Receiver operating characteristic (ROC) curves. (**A**) Homer2 and Homer3 for HCC vs Controls; (**B**) Homer2 and Homer3 for HCC vs HBV; (**C**) Homer2 and Homer3 for HCC vs patients with cirrhosis; (**D**) the combination of Homer2 and Homer3 for HCC vs Controls; (**E**) the combination of Homer2, Homer3 and AFP for HCC vs Controls.

To assess whether Homer2 and Homer3 could be used as potential diagnostic markers for HCC, ROC was carried out using 3 models: HCC vs Controls, HCC vs HBV, HCC vs cirrhosis (**Table 3**). Results showed that areas under the ROC curves of Homer2 and Homer3 to discriminate HCC patients from the controls was 0.743 and 0.798 respectively (**Figure 5A**). However, the diagnostic value of Homer2 and Homer3 for discrimination HCC from hepatitis B and cirrhosis was not obvious (**Figure 5B-C**). Combination of Homer2 and Homer3 possessed a moderate ability to discriminate HCC patients and controls with an area under the ROC curve of 0.809 (**Figure 5D**), while the area reached up to 0.900 when they were combined with AFP (**Figure 5E**).

## Discussion

HCC is a public health burden due to its relatively high incidence and poor prognosis 20, 21. Studies have reported that over 50% of HCC cases are caused by persistent HBV infections 22. To improve the prognosis of HBV-related HCC, it is of great importance to identify new appropriate biomarkers to diagnose HCC. Currently, AFP has been widely used for the diagnosis of HCC, but the results are far from satisfactory owing to the limited sensitivity and specificity 23, 24. Therefore, it is pivotal to explore new biomarkers to diagnose HCC at an early stage. Homer is well known as scaffold proteins 25 and plays an important role of in keeping the homestasis of calcium in intracellular 26. Wu and his colleagues validated Homer1 was upregulated in intrahepatic cholangiocarcinoma than that in the adjacent tissues 27. Our previous research showed that Homer1 could be a novel biomarker for HBV-HCC 17. It's logic and interesting to study whether the other two family member Homer2 and Homer3 can be new biomarker for HCC.

In our study, we revealed that Homer2 and Homer3 were downregulated in HCC tissues comparing with the adjacent noncancerous tissues. After analyzed the association between these two markers and clinical characteristics of HCC patients, we found that Homer2 expression was associated with tumor differentiation grade and TP level, while the expression of Homer3 was significantly related to tumor size, tumor nodes and GGT level. As we all know, an ideal clinical biomarker should be noninvasive and easily accessible. Thus, we subsequently tested the expression of Homer2 and Homer3 in peripheral blood leukocytes. In accordance with the results from tissue, the expression of Homer2 and Homer3 in HCC were both lower than that in healthy controls. By following up study, we found the increased expression level of both Homer2 and Homer3 after operation, indicating their potential value as surgical effects markers. At last, we identified that the combined AUC of Homer2, Homer3 and AFP reached up to 0.900.

In our present study, we found the expression of both Homer2 and Homer3 were downregulated in HCC, which indicated a possible tumor suppressive role of Homer2 and Homer3 in HCC. In accordance with our study, Mhawech et al. 16 concluded that patients with higher Homer2 protein had a better outcome in endometrioid endometrial adenocarcinoma. However, other studies have validated that Homer might promote the process of tumorigenesis. Sun et al. 15 revealed that the expression of Homer2 was significantly correlated with the markedly reduced overall survival of RC patients. With evidence supporting a tumor suppressor role of Homer in some contexts but an oncogene in others. We therefore think that Homer may function differently in different types of malignancies.

It is well known that HBV infection causes a rapid immune response, causing a life-long immunity with acute self-limited infection in more than 95% patients 28 and more than 50% of HCC are attributed to HBV infection 29. Studies have validated that inappropriate cytotoxic T lymphocyte (CTL) response triggers cumulative hepatocyte damage to induce chronic inflammation and ultimately develop into HCC 30, 31. Huang et al. 18 found that Homer2 and Homer3 could negatively regulate the activation of T cell. Besides, Homer-3 was found to inhibit the activation of serum response element (SRE) in T cells 32. Thus, we deem that Homer2 and Homer3 may suppress the progress of HCC by inhibiting the activation of T cells. This may be a research point in our future studies.

In summary, Homer2 and Homer3 expression were downregulated both in HCC tissues and peripheral blood. The expression of Homer2 was associated with tumor differentiation grade and TP level, Homer3 was related to tumor size, tumor nodes and GGT level. In addition, the combination of Homer2, Homer3and AFP could differentiate HCC patients from healthy controls effectively. These findings firstly indicated that Homer2 and Homer3 might be useful makers to diagnose, even to monitor the surgery effect, Further studies in a large number of patients are required to confirm the usefulness of Homer2 and Homer3 in HCC.

## Figures and Tables

**Table 1:** Clinicopathological relevance analysis of Homer2 and Homer3 in HCC patients.

Characteristics	n	Homer2 relative expression (log)			Homer3 relative expression (log)		
		Mean ± SD	t	*P*	Mean ± SD	t	*P*
Tissue			6.072	0.0001**		3.011	0.004**
HCC	77	-1.85±0.83			-2.27±0.62		
Adjacent Tissue	77	-1.37±0.72			-2.11±0.54		
Gender			-0.597	0.552		0.583	0.562
Male	71	-1.83±0.86			-2.29±0.48		
Female	6	-2.04±0.39			-2.16±0.76		
Age			1.419	0.160		-1.212	0.229
<55	39	-1.98±0.82			-2.20±0.50		
≧55	38	-1.71±0.83			-2.34±0.50		
Alcoholism			1.258	0.214		-0.031	0.975
Negative	38	-1.89±0.83			-2.31±0.49		
Positive	18	-1.56±1.05			-2.32±0.52		
Differentiation			-2.594	0.012*		0.960	0.340
High/ Moderate	58	-1.72±0.82			-2.30±0.50		
Low	9	-2.47±0.7			-2.12±0.54		
Size			-0.332	0.741		-2.67	0.010**
<5 cm	21	-1.75±0.84			-2.04±0.47		
≧5 cm	47	-1.82±0.86			-2.38±0.49		
TNM stage			0.001	0.999		-0.347	0.730
Ⅰ~Ⅱ	25	-1.75±0.7			-2.3±0.57		
Ⅲ~Ⅳ	29	-1.75±0.79			2.35±0.46		
Tumor nodes			0.447	0.657		2.312	0.026*
Single	31	-1.87±0.97			2.44±0.44		
Multi	8	-1.7±0.94			-2.05±037		
Cirrhosis			0.702	0.487		0.987	0.330
Negative	10	-2.08±1.22			-2.34±0.59		
Positive	29	-1.82±0.93			-2.16±0.25		
AFP (ng/l)			-1.509	0.136		1.480	0.144
<200	33	-1.67±0.61			-2.35±0.43		
≧200	32	-1.98±0.99			-2.17±0.54		
GGT (U/l)			0.126	0.900		-2.618	0.001**
<55	28	-1.81±0.66			-2.10±0.45		
≧55	42	-1.78±0.95			-2.40±0.49		
TP (g/l)			-2.195	0.032 *		0.950	0.345
<65	22	-1.51±0.64			-2.37±0.51		
65-85	47	-1.96±0.87			-2.24±0.49		
Alb (g/l)			-0.520	0.605		-0.319	0.751
<35	12	-1.68±0.45			-2.24±0.55		
35-55	57	-1.82±0.91			-2.29±0.49		

Data are shown as mean ± standard deviation. Since we failed to collect all characteristics of the patients, the total number may not be 77. TNM, tumor-node-metastasis. AFP, α-fetoprotein; GGT, γ-glutamyl transferase; TP, total protein; Alb, albumin. * *P*<0.05, ** *P*≤0.01.

**Table 2:** Characteristics of the studied participants.

Characteristics	HCC	Cirrhosis	Hepatitis B	Control	*P*
	N =72	N =59	N =52	N =109	
Gender					0.259^a^
Male	59	49	37	80	
Female	13	10	15	29	
Age					0.216^a^
<55	26	27	16	54	
≧55	46	32	36	65	
Smoking					0.198^a^
Negative	38	39	27	70	
Positive	34	20	25	39	
Alcoholism					0.764^a^
Negative	45	38	35	76	
Positive	27	21	17	33	
ALT (U/l)	41 (27,70) **	27 (19,38) *	79(31,283) **	20(17,27)	<0.001^b^
AST (U/l)	50 (31,97) **	36 (24,45) **	50 (31,90) **	22(19,26)	<0.001^b^
TBIL (μmol/l)	21 (14,27) **	22 (14,36) **	21 (13,79) **	13 (11,16)	0.002^b^
GGT (U/l)	147 (61,296)**	40 (22,104)*	54 (20,109)* *	22(18,35)	<0.001^b^
GLU (mmol/l)	5.1 (4.6,6.0)**	4.6 (4.2,5.9)	4.6(4.2,5.4)	4.4 (4.7,5.3)	0.002^b^

Data are shown as Median (25 Percentiles, 75 Percentiles).* *P*< 0.01, ** P< 0.001 *vs* Control. ^a^Chi-square test; ^b^Kruskal-Wallis. Abbreviation: ALT, alanine aminotransferase; AST, aspartate aminotransferase; GGT, γ-glutamyl transferase; GLU, glucose; AFP, α-fetoprotein.

**Table 3:** Comparisons of the AUC of the expression of Homer2 and Homer3 for subgroups.

Group	Gene	AUC	95%CI	*P*	Se (%)	Sp (%)
HCC vs Control	Homer2	0.743	0.671-0.814	<0.001	61.5	77.8
	Homer3	0.798	0.730-0.863	<0.001	65.1	87.5
HCC vs HBV	Homer2	0.696	0.605-0.789	<0.001	96.2	36.1
	Homer3	0.698	0.606-0.790	<0.001	80.1	56.9
HCC vs Cirrhosis	Homer2	0.691	0.601-0.780	<0.001	86.4	45.8
	Homer3	0.691	0.600-0.782	<0.001	83.1	47.2
HCC vs Control*	Homer2, Homer3	0.809	0.744-0.874	<0.001	72.5	83.3
	Homer2, Homer3, AFP	0.900	0.838-0.963	<0.001	83.3	83.9

**Abbreviation:** Se: Sensitivity; Sp: Specificity; AFP, α-fetoprotein. * Combination of Homer2 and Homer3 or Homer2, Homer3 and AFP to differentiate HCC patients from the controls.
